# Suppression of Gonorrhœa

**Published:** 1887-08

**Authors:** 


### SUPPRESSION OF GONORRHŒA.

During last meeting of the I. H. A., a paper was read showing the evil results which follow the suppression of gonorrhœa by injections, etc. A most interesting discussion ensued, which we give here:

Dr. Lippe—There is no doubt about the correctness of the position taken in the paper, "Gonorrhœa can Kill." There have been some cases in Philadelphia. The patient I would like to allude to was a young gentleman, highly educated—a fine man. He has been a patient of mine since his birth. I have waited upon him in all sorts of diseases, small-pox, typhoid fever, and colds, and he has always recovered. He was a strong homœopathist, but he fell into the hands of some of his "lady friends" and became ill, and he was ashamed to go to his old doctor. So he called in the Philistines and they made injections. His gonorrhœa stopped promptly, but he became afterward hoarse, and his hoarseness increased. I was not consulted, and I don't know who advised it, but his father went over with him to London to consult Mackenzie. Both this young man and his friend, the Crown Prince, fell into the hands of Mackenzie the same way. Mackenzie took off something from his larynx and then sent him home. He got better, but whatever that was, whether it was a wart or something else, which I think it was, it came back again. It was taken out again. It never grew again on that spot, but the gentleman became sick and has been sick for the last six or seven years under my care. I have never been able to cure him. I have from time to time put the brakes on and got him better, as the disease is probably of a cancerous formation. Now he has moved out of town in the neighborhood of my friend, Dr. Clark, who is treating him and keeping him comfortable, and will see him off easily. But the treatment applied by the so-called homœopathic school, by the *quasi*-homœopathic pretenders, brings forth more evil than any other line of treatment of any kind in any disease. Whether we can ever cure these unfortunate patients who have fallen into the hands of the injection party I do not know. I have had a case that I have apparently cured, but wait awhile, wait at least seventeen years, and at the end of that time—I have known it at the end of that time, like the locust, to come back again.

Dr. Bell—Gonorrhœa and syphilis?

Dr. Lippe—Gonorrhœa and syphilis. It makes no difference. It has a manifest time of seventeen years. In seventeen years it will come back again and you get the same symptoms. When he gets ill again he will never get well. I have not cured such a case yet. With the greatest care you take I think the disease comes out again after being latent that long time.

Dr. Bell—After bad treatment?

Dr. Lippe—After bad treatment—unhomœopathic treatment. With good homœopathic treatment I have never seen a particle of stricture, and never seen any swelling of the testicles, and I have never seen any evil results. They get well, but they do not always get well in two or three days. The common catarrh, which you have the credit of curing, gets well in a week's time easily; but if you get a sycotic case of gonorrhœa you will thank your stars if you cure it in six months. The people will have to wait, and if you have any patients you will have to tell them so. After they have been treated by the old school they never get well. That has been my experience for many years.

Dr. Brown—Mr. President and members of the Association, I wish to put in an idea here which may help you in curing such cases, having had experience in two cases, which I did not lay so much to gonorrhœa as to the previous disease the patient had. My experience has been that whenever a man is taken with that disease, if he has any other disease about the system, it will take from thirty-six to sixty days without any local application—nothing except cleanliness, no internal treatment of any kind. Avoid exercise, and keep the system in proper condition, and it will all disappear. Then select the best homœopathic remedy according to the symptoms of the patient. That has been my success, and will be your success if you follow that plan. I could not cure a case that is complicated with some other disease of a serious character, and I think others have found they cannot cure under such circumstances. I do not want to lay so much to the gonorrhœa as to the complication of diseases which we will find many cases have already in that way.

Dr. Wesselhœft—In answer to what Dr. Brown has just said, we all, I suppose, use cleanliness and care and pay attention to the diet. My experience with this disease has not been very large, but it has been large enough to draw some conclusions, but I can only second what Dr. Lippe has said in the course of the treatment of acute gonorrhœa, sycotic and non-sycotic. All these acute symptoms, however carefully the case may be treated, may arise, and I mean by that that the sycosis may come out and sycosis will recede, and there will never be or should never be any chronic enlargement of the testicles. As regards the preceding diseases, of course my personal experience has not extended over the time that Dr. Lippe's has, but I know from the experience of my father, and from my experience with people who I know have been treated by my father for that disease, and I know also a few instances of where men have arrived at the age when prostatic diseases come on, and from those instances I can infer that with perfectly true, honest administration of homœopathic medicines a prostatic disease, consequent upon an early gonorrhœa or orchitis can be cured. Chronic cystis is one of the very great exceptions in our school—I mean in the Hahnemannian school. I have had instances that have interested me very much, as of the repression of old suppressed gonorrhœa. I now recollect three of those instances where I am perfectly sure that there were no infections. One of them occurred seven years after the suppression, and it is so interesting a case that I will here outline it. Of course, it can only be outlined, as I am not prepared, and was not prepared, not thinking that I should be called upon. A very long, lank New Englander, who was about six feet three inches high, came into my office, sprawled himself down on a chair, and said, "Can you cure dizziness?" I replied that I did not know whether I could cure dizziness, but asked, "What is your dizziness?" He said, "I have been dizzy at times for six years. I have been taken up several times for drunkenness in the street, and it came on after a fall from a wagon. I was driving an express wagon, and my horse or something jerked, and I fell between the horse and the wagon, receiving a blow on the head." That was about the history of the case. The characteristic was vertigo. I can recollect that without notes. I treated the man, but the treatment was without the slightest benefit. Finally, it was long afterward, he was in my office one afternoon, and I was having a confidential talk with him, and I re-examined the case with him as well as I could. He denied that he had ever had any disease of any kind—skin disease—and I asked him in my usual way of examination: "Have you ever had any syphilitic infection?" and he said, "No." On this occasion he told me, "You never asked me if I had had the clap, but I have, and I wonder whether that could have had anything to do with it." I said, "No doubt of it." "Well," he said, "I had it when I was thrown from the wagon, but the day before I was thrown from the wagon I had an operation performed that was the most painful thing that I ever knew of in my life, and I went to bed in the afternoon and howled." He said, "You remember that old homœopath in Salem by the name of Flotar"—you will remember him, Dr. Wells.

Dr. Wells—Yes, sir.

Dr. Wesselhœft—"He said, 'I will stop that for you,' and he took a stick of Lunar caustic, and he put it in and he pulled it out. Well," he said, "I was so sick that I could hardly walk or do anything, but I had to go on to my wagon and drive." Mind you, this was about four or five months after I had begun treating the man. Of course, my first idea was, after I had heard this story, that the man got dizzy and fell from his wagon, and that there was no jerk about it at all. I gave him Thuja, and in ten days he came back to me, and I shall never forget his entrance. He says, "Doctor, now I am going to be truthful with you. There has been no new infection, but I have got my gonorrhœa back again." Well, there was a cold shudder went down my back. I could not tell what it was caused by in so short a time, and I asked him. He said, "That is all I know." I told him I must know the reason why, and he said, "I am telling you the truth, Doctor, and there is no reason why I should not be perfectly open with you, and I assure you that there has been no infection, but," he said, "I have not been dizzy once this week." "Well," I said, "let us go on." He got Sugar of milk, and he did go on and he had, to all intents and purposes, the most acute, and actively acute, gonorrhœa for six weeks that I ever saw. I treated that man for eight months and I did not stop the gonorrhœa, but in those eight months that man became so convinced of the fact that gonorrhœa was the base of the trouble that he left me and went out West and went to running a locomotive.

Dr. Brown—I wish to say one word with reference to cleanliness. Now, I did not mean that all physicians do not use or think as much of cleanliness as I do or that all physicians do not try to correct the habits of their patients in regard to the use of liquor, tobacco, and coffee, and those things which prevent the cure of gonorrhœa, but I want this to be understood, that I found this to be an easy thing when I could get my patients to leave off their liquor, tobacco, and coffee, and stimulants, and live plainly on bread and milk, that I could cure them in so many days, and I can keep my word with them. If a man is otherwise healthy I can do it. In regard to the rest of the matter, as Dr. Wesselhœft has said, I think as physicians we are all level-headed. We pay attention to diet and all the other circumstances as we would do in acute diseases.

Dr. Gee—Dr. Ballard has an interesting case which would confirm Dr. Wesselhœft. I would be glad to hear his report of that case if he will present it.

Dr. Ballard—I am hardly ready to report on that case, but I can say a word or two that will show that the matter is progressing favorably. It was the case of a young man, who, a number of years ago, was living in Germany as a student. It was five or six years ago. Previous to that, I believe, it is said that he used to be troubled with skin affection, a little eruption appearing here and there, and then he contracted gonorrhœa. Now I am not certain, because I have not my notes here, whether this affection appeared subsequently to gonorrhœa or not, but then came the time when this was stopped, suppressed mostly by injections, and he supposed that he was cured. Then came on an attack of rheumatism. I will speak of this. It began in the feet and extended up to the joints, and soon he had iritis. I will give you the terms as I got them. A physician treated the patient for rheumatism. Then came the ophthalmia. Now the iritis. He said, "I cannot manage this case alone; I must have some help. I must have an eye specialist." So one of our prominent men was called to prescribe for the iritis, and the other doctor prescribed for the rheumatism. The patient was a very intelligent man, and he said, "The eye man came to see me yesterday and he gave me Mercurius for my eye, and to-day my physician came to see me and he gave me Pulsatilla for my rheumatism." And he swung along in that way when I called to see him. To make a long story short, I will simply say the eye trouble disappeared first, and it got down to his knees and got down so that he could go around "clump-footed" in this way, and I kept on. Then his physician returned; he had been away. I told him, "Your physician has returned now," and I notified the physician of the fact that I had been treating his patient and I said, "I resign the case," and then he passed away into the hands of the other physician and he went along. After a time he got to clumping again in this way, and the first thing I heard he was confined to his house with ophthalmia. Then I was called to take charge of the case on the second day of last January. I found him pretty well lamed up and one eye in a very bad state, granulations appearing; unable to bear the slightest ray of light, a great deal of pain darting around through the head, etc. The other eye was very sensitive indeed to the light. I took a very careful note of the case and gave him a dose of Psorin. 42M on the second day of last January, he having the gleet, as I said, and he has had no medicine since then, not a whiff of anything, and he is tramping around just as free as ever. He don't mind the storms, and he has not a vestige of the rheumatism about him, and he has not a vestige of his gleet, and no trouble at all. He considers himself perfectly well in every respect, and only that one dose was given to him. The gleet, I will say, being the first thing, has only recently ceased to show itself and he is not having any of it. Now, I was hardly ready to report the case because I wanted to wait until another session, but as I have been called upon I will give it that far.

Another case was that of a young man who came into the office, and he had dizziness, headache, and a sort of eruption about his eyes occasionally, and a great deal of thirst. I cannot recollect the symptoms fully, but I asked him questions and found that he had had the clap. It was cured seven years before. I prescribed for him, the remedy being, I think, Argentum nitricum$^{35m}$. He returned to me in about ten days and said that he was having a discharge from the penis, and he was a truthful negro, though he was black. I have known him pretty well since, and I am perfectly satisfied that he told me the truth. There was no inflammation, no indication of acute trouble in any shape, and he went along in that way; the gleet gradually ceased, becoming very much better. One of the peculiarities of his case, I remember, was the pain in the head, and he complained of it a great deal. I got his symptoms as best I could and prescribed for him; and he would have attacks of great thirst, usually in the latter part of the day. He would drink enormous quantities of water. I asked him if the water made him feel any better or any worse, and he said that he thought it made him feel a little better. After he drank, if the water was very cold, he would feel badly about the head. I could not get that symptom fully, but after prescribing for him for a number of weeks he went along and I could not control that pain in his head. But one time he told me that after drinking cold water this pain would seat itself in the forward part of the head and extend down into the nose. This was a symptom under Digitalis. I gave him one dose of the 2M, and that wound the trouble up. [Applause.]

Dr. Nash—I have learned this in the treatment of this disease, and I think a good many physicians have also learned the same thing, and that is, in cases of gonorrhœa not to suppress the gonorrhœa, that bad effects follow from its suppression. This fact has been lost sight of. There is a certain class of remedies that are commonly used in gonorrhœa, and they apply them for that reason. I have cured these cases of chronic gleet that have been running for months with remedies that I never had used for it, and that I never have found any one else to have used for it, simply on the indications that were presented at the time when the patient came to me. I remember curing one of the worst cases of gleet that I ever had or ever saw with Kali hydriodicum. There are other remedies which were also used. There was a patient who came into my office two months ago—and, by the by, I want to say this—that young man came into the office and wanted to know how long it would take to cure him. I always explain to patients when they want to know how long it will take to cure them, and I always tell them frankly, that I do not know. I do not know whether it would take me six days, one month, or six months to cure, but you pay me your money, so much money now, and I will cure you if it takes me a year to do it. There will be no more charges. I take the responsibility of this case from now thenceforth. But if we can only make our patients see the fact, and there are plenty of instances that we can bring to prove these facts that the allopathists would in many instances even suppress. The first thing they do is to suppress the discharge, and that is the worst treatment that could be practiced. Where they were not even able to suppress the discharge that was running into chronic gleet, they used injections to suppress the discharge. This is bad treatment. Two months ago I had a case of this kind come into my office. He said to me that a certain physician was treating him. The physician was a fashionable gonorrhœa doctor of the place, because he cured them quickly, and he always did it with injections. With what result? They have something that has lasted for years. This patient to whom I have referred has a discharge of slimy mucus, from ten to fifteen drops, or a tablespoonful. It looks like milk. This is undoubtedly a discharge from the prostate gland—prostatic gleet. His symptoms were covered with Selenium. I gave him one dose of Selenium$^{40m}$, Fincke, and Sac. lac. and to follow directions and return at the end of the week. At the end of the week he returned and said, "Doctor, I have had only one discharge of that kind since I took that dose of medicine!" Two weeks after he returned and that was the end of the whole business. [Applause.]

Dr. Wells—In one respect, in reference to gonorrhœa, I stand very much as my friend who spoke first, as I have not had an extensive practice, but I have had more than I want of it. I have treated a good many cases from first to last, but never with anything but the specific remedy, and in no case, in now nearly fifty years, have there been any sequelæ. They have been cured. I did not rise so much to mention that as I did to call attention to the consideration which we all ought to take of the cases related by our coming President. We are all supposed to be familiar with the *Organon.* That *Organon* gives the instructions of the grand old man in relation to suppressing local manifestations of the disease, and a grander confirmation of the instructions of the *Organon* than that I have not heard for many a day. I have met more than has been pleasant from the consequences of suppressed local manifestations of disease; in general destructive processes result; and now my object in getting upon my feet is to impress upon this company, and on all my associates, that a suppressed gonorrhœa is a suppressed inferno, and to me the characteristics which our old-school enemies annually recommend are simply the result and not the disease at all, no more than what you gather in the handkerchief in influenza, is the influenza. You have simply shut up their exit to the ventilation of the organism of the disease. You have stopped it up and shut up the outlet—shut up the disease in the organism itself. There it will work a work of destruction to the end unless it finds the specific remedy. I have had more experience of this sort, and more patients on my hands than has been pleasant, for there is no more difficult thing to master and to cure than the results of suppressed local diseases of this kind. I hope it will go to my neighbor and not come to me. I have seen in consultation, within the last four months, a man who had been ill for eleven years. He did not know what was the matter with him, and when he was explaining what was the matter with him, he said that eleven years ago he was in trouble, but there has been nothing of the kind since—nothing at all. He was cured by the old-school treatment, but he had not seen a well day. He had been sick all those eleven years. That man got one dose of Thuja$^{40m}$. He was another man in a little while. The particular localization was manifest. When he came under my attention he had a peculiarity of the urinary secretions which had been for years, not exactly a sediment, but a material opaque body that floated in the urine. It was not deposited, it looked almost like milk thrown into the urine, only the particles of white matter were larger than milk globules. This all ceased in a few days after that. He was only under my care for consultation, and this was the result of my consultation. He was suffering from stricture, and he was cut for stricture, and that is the last I know of the case.

But this suppressing by local application is all wrong. Just listen to the old man and be the wiser for it as long as you live, and it is true, whatever else is or is not.

Dr. Schmitt—At the same time I will say that there is no necessity for suppressing the gonorrhœal discharge, because anybody knows we have two different gonorrhœas. One is situated in the canal, which is a common urethritis, just like a common coryza, and it will get well by itself often if a person does right and gets the right homœopathic remedy; the other is sycotic. I maintain, and I suppose that a good many of you share my views, that we have two gonorrhœal affections, just as we have two chancroid affections; that is, urethritis cases, and also those properly called chancroid and sycotic cases. This urethritis comes, perhaps, parallel with chancroid, and sycotic cases come parallel with chancroid, just the same as you may cure chancroid with Mercurius, while the real sycotic gonorrhœa, which manifests itself in an entirely different way from urethritis, you will always get an anti-sycotic remedy, even so far we have a parallel between chancroid and urethritis. Chancroid comes on perhaps three, four, or five days after infection, and urethritis comes on after the ninth day and generally even earlier, but it will never have the constitutional symptoms with it then. Chancroid comes on after three weeks.

Sycotic gonorrhœa comes on after three weeks, and the patient is sick before the discharge appears. He feels sick, and then comes the discharge that has to be treated ; so the whole organism is affected, just as in chancroid the whole constitution is affected before the chancroid manifests itself. If, for instance, our urethritis is suppressed or maltreated, of course psora will come up if there is no other chronic trouble in the case; then you have to treat the results—the gleet—by antipsoric remedy. In sycosis you have the sycotic symptoms, and you have to treat the sycosis.

Dr. Biegler—A suggestion that has come to me in listening to the discussion on this subject is that we might look a little further for the mischievous influence of this suppressed disease. I mean it comes in concealing language in our professional lives. It is well, like good navigators, to mark in advance the rocks upon which we may strike. We have considered the subject of suppression in an individual. Now I have come to think and believe that whether a disease is cured or suppressed in an individual that the mischievous influences, the results, are not likely to end there. In the case of married life I have reason to believe that the wife, especially if she has borne children from the husband who has been poisoned, who carries with him that poison, that the wife also suffers in an unknown way. I say unknown because it has never been appreciated. Many of the ills of the wife and the children are the consequence of that disease. We may have general ill health, especially and probably in the way of uterine difficulties, catarrh, and so on, that if we can only bear in mind the probabilities in those cases, we may yet do a good deal of good by prescribing in accordance with that view. I have reason to believe that many families, wives and children, are ruined, and are given very impaired constitutions from this cause.

Dr. Sawyer—I have had quite a number of cases of suppressed gonorrhœa to treat, but what I wish to speak of, more especially since sycosis has been mentioned in relation to these cases, is that I think it is exceedingly important to remember this fact, and the fact that there are so many more soft cancers, and so many more cancers—more especially of the soft variety—lupus exedens—than there used to be. There is a great deal more soft cancer; and I believe soft cancer—lupus—is always the effect of sycosis, and nothing else.

Dr. Hawley—I don't know whether this is a suitable occasion or not, but I would say that I have listened to this discussion with a great deal of interest, and I am inclined to speak of my own experience somewhat. I never have had a very great experience of gonorrhœal cases or syphilitic cases, but in my treatment of either I have sought only for the specific remedy. I have never had a case of gonorrhœa that I have treated in its incipiency that was followed by any sequelæ of importance. I never had, with one exception, a case of fig-warts, for instance, as a result of gonorrhœa that I had treated. I have been greatly annoyed sometimes with the persistence of my cases, and I always tell the patient, as some one else said—Dr. Nash, I think it was—that I do not know how long he will be sick, but yet, if he don't lose his patience, he will get well; but I have had one notable exception. I may say that I never had but one case of syphilis or chancroid that was followed by any secondary symptoms, in all my experience as a homœopathic physician, but I had one very notable case, and I want to speak of it, because it bothers me. The patient came to me with what I supposed to be a chancroid. It had been neglected eight weeks. He had done nothing for it. It was about two weeks after he came to me when the chancroid disappeared, and with it appeared a syphilitic sore throat that soon disappeared, as I supposed, under the action of my remedy; but it was scarcely out of sight before he was troubled with syphilitic maculæ from the crown of his head to the soles of his feet. In a style that was still more prominent and marked, he took the syphilitic iritis, and I thought for about two weeks that he would certainly lose the right eye, and I was afraid of his losing both, but he came out of it with both eyes. That was followed about three or four weeks later with the most terrific neuralgic pains in the head that I ever saw in my experience. This lasted nearly three weeks. Still the maculæ were just as marked and prominent as they had been.

Dr. Bell—What about the chancre?

Dr. Hawley—The chancre disappeared about the time the sore throat did, and without any local application. Very soon after this neuralgic pain in the head commenced his hair and beard began to fall out, but as this progressed he apparently recovered a pretty comfortable degree of health and the maculæ disappeared gradually. I felt greatly impressed, and thought I was doing pretty well, considering the length of time he had been without any treatment. He got so that he resumed his business, and about the only symptom he complained of was a sense of "don't care." He did not care whether his business went on or not. He started in the morning, thinking he was going to do a day's work, and sat down and did nothing. He remained that way for more than a year. He came to me with three ulcers behind the glans, which I was disposed to regard as chancroids. They were attended with the most profuse gonorrhœal discharge that I ever saw in my life. I accused him of a second exposure; he denied it. At this time I asked the question, which I had never asked before, how long after the first exposure was it when the chancre appeared. He was very definite and positive in his statement that it was just exactly eight days. Now, there are some who teach that secondary symptoms never follow a chancroid, but a chancroid always had the period of six or eight days' incubation.

Dr. Bell—Had he not had any infection before that?

Dr. Hawley—He said not. He was a personal friend, and I took his word where I would that of any man. He said he never had had but one connection. I learned afterward before the symptoms appeared that he had probably made a mistake.

Dr. Bell—He had probably had two.

Dr. Hawley—Yes, sir, he probably had had two at least. I meant to use that word as a noun of multitude. I went at him again, with my best endeavors, and in a very short time I had the satisfaction of seeing this profuse gonorrhœal discharge disappear. The chancroids, if they were chancroids, healed and he became fairly well again. Still he kept under my treatment, but I seldom saw him; he lived two hundred miles away from me. But for about two years and a half he was in New York—it is about seven months ago—and fell in the street with partial paralysis of the left side; he was taken up and recovered enough to be taken to the train and sent to Syracuse. His face was drawn quite a little to one side, his speech was considerably affected, and his ability to walk so much impaired that he was disposed to walk around the left leg, and he would walk in a circle; he would walk against the side affected in the space of six feet, and would come around to the place from which he started. I prescribed for him that night when he got there, arriving in the evening. The next morning I thought his paralysis very much better, so that he walked fairly well. I tried to have him stay in town, but he did not, he went away. He did not go home, but went to his mother's. Three days later he was prostrated with paraplegia, complete loss of motion and sensation in the extremities, and there he lies yet. There is one thing very remarkable about this to me, and that is, that it was neither chancre nor chancroid, according to the definition.

Dr. Ballard—I will speak of just one case which shows most decidedly the evil effect of suppression. I think the most important is to ask the question as to whether a person may show syphilis without the new secondary form.

Dr. Bell—The subject is gonorrhœa and not syphilis.

Dr. Ballard—I was going to speak of the suppression of the discharge in which there came on melancholia and a disposition to end his life. He fought against that for a long time. For a long time he was melancholy all the time, and yet after his great fits of melancholy there came a brightness, and everything looked bright; after he passed out of one of these spells everything looked bright. His appearances all looked quite good to me. He liked to see his friends, and he seemed to be so happy; as he passed from that condition there was a settled melancholy and a disposition to end his life by shooting. I gave him a dose of Sulphur to begin with. That was before I learned the trouble and the disposition to kill himself. I did not treat that man but a little while before he came to me again with another attack, and he said he had not been exposed, and every indication was of an acute trouble, and that readily yielded to treatment. I don't know what it was. The man passed out of my hands, but would not pay his bill.

The President—That is the interesting part.

Dr. Bell—This paper is so important and valuable that I don't want to leave it, but I suppose we must. I wish very much that all of our young men could have heard the discussion this morning, and the young men outside of the Society. It is more than true that gonorrhœa can kill. Of course, as Dr. Hawley says, it is the treatment that kills. It is the treatment that we may say kills in the gonorrhœa practice, but they get the treatment, and, therefore, the gonorrhœa kills. I wish all the young men to know that the allopathic authorities say that gonorrhœa kills, not only that, but that it is more fatal than syphilis, although it seems to be so slight a disease, according to their own statement, a local disease to be suppressed, and yet they also declare, and this is by their best authorities, that gonorrhœa is more fatal in its results than syphilis. It is dangerous when stricture exists and secondary symptoms which they all recognize, and that is what I wish all young men to know and to understand. I wish they could all read and understand the best authorities. Otis, of New York, decries the use of injections. Ingram also expressly denounces the use of horrible mixtures used by the old school. He expressly lays down the doctrine which Dr. Schmitt has stated and Dr. Lippe has also mentioned. He says "your injections will cure your urethritis, but will not cure your gonorrhœa." He says so. That is the very thing we want to know. There is no doubt about it. As Dr. Nash has said, we must take plenty of time to cure them. They must be cured and not killed. We may add that it will pay to take time. Most people are capable of receiving such instruction and profiting by it. It is worth while to take time, and you may say that it pays to know what is being done by others. They tell you, and the young man's friends tell you, that they can get cured in three days, and all this kind of thing. So they do. They will tell you all that kind of thing, and your answer must be all that you propose to do is to cure them thoroughly and radically of the results of the terrible disease.

